# Supplementary material for: Are carbon emissions trading and green financial instruments synergistic? -Comprehensive quantitative research based on content analysis
Source: PLoS One. 2024 Mar 7;19(3):e0298601. doi: 10.1371/journal.pone.0298601 (PMC10919601; doi:10.1371/journal.pone.0298601)
Supplement: S1 Data — (ZIP) [file pone.0298601.s001.zip › Data/地级市Z_F工作报告.zip!/┴╔─■/│»╤⌠╩╨/└┤╫╘╣1⁄2╓┌║┼í╛┬φ┐╦╩2╛▌═°í┐_2011.docx]

各位代表：

我代表市人民政府向大会报告工作，请予审议，并请各位政协委员和其他列席人员提出意见。

“十一五”规划完成情况及2010年工作回顾

“十一五”时期已经结束。五年来，在省委、省政府和市委的正确领导下，我们与全市各族人民一道，深入贯彻落实科学发展观，抢抓机遇，顽强拼搏，克服了国际金融危机、历史罕见旱灾等不利因素带来的严重困难，全面实现了“十一五”规划目标，改革开放和现代化建设取得新的令人瞩目的成就。

——经济实力快速提升。全市地区生产总值年均增长19%，高出规划目标2个百分点，总量是“十五”末的3倍；地方财政一般预算收入年均增长35.7%，高出规划目标17.7个百分点，总量是“十五”末的6倍；固定资产投资累计完成1773亿元，年均增长47.5%，高出规划目标17个百分点；社会消费品零售总额年均增长18.7%，高出规划目标1.5个百分点。2008年以来，全市地区生产总值翻了一番，地方财政一般预算收入和固定资产投资分别翻了一番半，圆满完成了“奋战三年、再造朝阳”的战略任务。

——改革开放继续深化。国有企业产权制度改革全面完成，民营经济实体迅猛增加，一大批企业通过体制机制创新释放出巨大的发展能量，民营经济增加值年均增长45.6%。外向型经济全面推进，累计利用外资2.6亿美元，引进内资1100亿元，分别比“十五”增加9倍和8倍。

——产业结构不断优化。一批重大项目开工建设，特色产业集群逐渐生成，工业化进程明显加快。现代农业和新农村建设迈出新步伐，农业结构深入调整，生产方式加快转变，产业化程度不断提升，农村经济呈现空前活力。城乡服务业实现较快发展。三次产业比由“十五”末期的26∶39∶35调整到22∶51∶27。

——城乡建设协调推进。新增公路里程8200公里，比“十五”末增长2.4倍，其中高速公路386公里，增长7.2倍；行政村全部实现通油路。累计投入城建资金330亿元，实施了一大批城市建设和改造工程，城市功能不断完善，城市形象全面提升。城镇化率由“十五”末的34.2%提高到42.8%。实施一系列生态建设和环境保护重点工程，森林覆盖率由“十五”末的33.5%提高到40%，城乡环境质量明显改观。

——民生状况持续改善。五年来，各级政府累计投向社会民生工程资金总额274亿元，占同期财政支出的57.1%。广开渠道安置就业30万人次，7600多户零就业家庭全部实现至少一人就业。养老、医疗、低保等社会保障体系逐步完善，保障范围不断扩大，保障功能明显增强。与改善民生、提高人民生活质量密切相关的教育、卫生、文化、体育等各项社会事业全面进步。城镇居民人均可支配收入、农民人均纯收入年均分别增长15.2%。

各位代表，过去五年，是全市上下开拓创新，实现历史性跨越的五年；是城乡面貌发生巨大变化，人民群众得到较多实惠的五年；是贯彻落实科学发展观，为未来发展奠定坚实基础的五年。五年的辉煌成就，激发了全市人民攻坚克难、奋发进取的昂扬斗志，也使全市人民看到了自己家乡加速突破、加速振兴、加速崛起的美好未来！

各位代表，刚刚过去的2010年为“十一五”发展划上了圆满句号。经过一年的不懈努力，我们全面完成了市九届人大二次会议确定的各项目标任务。预计地区生产总值实现640亿元，比上年增长18%，三次产业增加值分别实现130亿元、327亿元和183亿元，增长19%、20%和16%；地方财政一般预算收入和国地税收入分别实现66.3亿元和90.6亿元，增长31.7%和39%；固定资产投资完成570亿元，增长25%；社会消费品零售总额完成215.6亿元，增长18%；城镇居民人均可支配收入和农民人均纯收入分别达到13400元和6100元，增长15%和18%。

一年来，我们重点做了以下工作：

——强力推进设施农业建设。将省委、省政府支持朝阳设施农业大发展视为历史性机遇，动员全市上下开展了100万亩设施农业建设攻坚战。新开工设施农业建设面积72万亩，其中52万亩实现定植；调地工作全部结束。这项措施将给朝阳农业生产和农民生活方式带来深刻革命，对稳定农民收入起到重要作用。

——不断提高农业生产能力和产业化经营水平。不失时机开展人工增雨，全面加强大田生产服务，粮食生产获得历史最好收成，总产量达43亿斤。新建标准化畜牧小区917个，各类畜产品产量均以20%以上速度增长。农业产业化经营步伐加快，新增销售收入500万元以上龙头企业75户。

——进一步壮大工业产业集群。新能源电器产业基地纳入国家火炬计划，入驻企业累计达到50户，投资总额50亿元。各县（市）区新增产业集群企业90户，集群规模不断扩张。北票粉末冶金和除尘装备、朝阳县有色金属产业集群产值均突破百亿元。

——强力推动重点工业项目建设。鞍凌精品钢一期、浪马子午胎扩产项目部分投产，燕山湖电厂主体工程接近尾声，500兆瓦太阳能光伏产业链项目开工建设，沈阳远大新能源风力发电项目一期主体工程完工，北票富贵鸟铁精粉深加工等项目竣工投产。

——狠抓工业经济运行。加大扶持力度，帮助重点企业调整结构，开拓市场，保障要素供应，使凌钢、朝柴、浪马等骨干企业稳健运行。全市规模以上工业增加值完成265亿元，增长22%；新增销售收入500万元以上企业200户。

——积极推进服务业发展。各县（市）区服务业集聚区开始规划建设。商贸流通等传统服务业持续发展，新天地商贸文化城开工建设，华府万国五星级酒店即将竣工。旅游、保险、邮政、通信、电子信息、文化产业等现代服务业不断成长。金融服务得到加强，全年新增贷款77亿元。

——继续深化各项改革。企业联合重组取得新进展，民营经济海外并购实现零的突破。新一轮政府机构改革基本完成，乡镇机构改革和集体林权制度改革不断深化。财政管理体制进一步理顺，财税征管机制不断创新。

——大力推进对外开放。高密度开展大型赴外招商活动，引进域外资金400亿元，增长30%；实际利用外资1.1亿美元，增长50%；实现出口创汇4.1亿美元，增长10.8%。

——加快强县壮区步伐。县域经济三年倍增计划超额完成。五县（市）实现地区生产总值400亿元，同比增长28%；地方财政一般预算收入43.5亿元，增长35.5%。北票、凌源、建平和朝阳县地区生产总值均超过百亿元，其中北票、建平财政一般预算收入率先突破10亿元。采取多种措施支持城区经济发展，两区财政一般预算收入均超过3亿元。

——加大城镇建设和管理力度。中心城区重点工程进展顺利，凤凰新城初具规模，大凌河三期工程实现蓄水，珠江路大桥开工建设，4条新街路建设和6条老街路高标准拓宽改造全面完成，城市绿化亮化工程大面积展开。以喀左为代表的县城规划建设水平不断提高；15个村镇基础设施、50个村屯整治规划和300个村镇综合整治任务基本完成。全市共实施房地产开发780万平方米，同比增长25%；实施老旧小区环境整治改造40万平方米。加大城市综合执法力度，市容市貌得到改观。

——启动建设全市瞩目的两座新城。在中心城区北部规划40平方公里的燕都新城，采取封闭式管理模式开发建设，各项工作已全面铺开；积极争取省政府支持，朝阳县城正式开工建设。两座新城建设拉开了中心城市南展北拓发展新格局。

——全面加强基础设施建设。朝赤高速公路朝黑段建成通车，101线改造全线竣工，重点铁路工程项目正常推进，机场扩建工程高质量完成；凌源、建平城区应急供水工程全面启动，北票引白入北调水工程如期竣工；与辽河油田合作输入天然气工程开始启动。

——大力改善生态环境。加速推进500万亩荒山绿化，全年完成造林224万亩；启动实施120万亩坡地绿化工程，30万亩年度任务全部落实；大小凌河流域治理保护工作即将全面展开。

——积极推进节能减排。朝阳发电厂实现关停，全市五座污水处理厂投入运营，市区生活垃圾填埋场投入使用。全面完成了节能减排目标，城区空气质量达到国家二级标准天数超过90%。

——广泛实施各类民生工程。完成实名制就业5.9万人，城镇登记失业率下降到3.9%；社会保险覆盖面进一步扩大，各项待遇得到较好落实；实施整村推进扶贫村102个，完成移民搬迁740户，新增户有稳定收入项目5万户。新建农村安全饮水工程713处，解决了20万人安全饮水问题。城乡低保和农村五保对象供养提标工作全面落实。实施廉租房建设及补贴、经济适用房建设和农村贫困家庭危房改造，解决了部分困难群众住房问题。加强对供暖企业政策扶持和监管，供暖质量明显提高。投入资金9.2亿元，全面完成了向全市人民承诺的15件实事。

——促进科技教育全面进步。积极组织研究开发、推广利用实用技术，有针对性地引进各类人才，科技创新能力得到增强；继续培植高新技术产业，实现增加值63亿元，同比增长25%。启动市级义务教育均衡发展示范县区创建工作，新建农村九年一贯制学校38所；朝阳师专新校址建设即将竣工，工程技术学校一期工程交付使用；学前教育、民族教育和民办教育都有新发展。

——推进文化体育事业健康发展。新建成标准乡镇综合文化站14个；牛河梁国家遗址公园展示馆一号地点顺利建成，二号地点完成主体工程，“申遗”工作深入推进；我市参与制作的大型电视连续剧《中国地》在朝阳拍摄任务圆满完成。成功举办了第三届全民健身节，承办了8项国家和省级大型体育赛事；我市代表团在省第十一届运动会上创造佳绩，共获金牌151枚。

——全面加强卫生医疗和人口管理工作。卫生医疗基础设施不断完善，重大传染病得到控制，医疗质量和服务能力进一步提升。继续采取综合措施落实计划生育基本国策，人口出生率和自然增长率分别完成控制计划。第六次人口普查工作全面展开。

——下大力量强化社会管理，狠抓武装和双拥工作。深入开展信访工作效率年活动，积极化解影响稳定的社会矛盾，促进了社会和谐。强化隐患排查、检查和整改工作，加大打“非”治“违”和整顿关闭力度，安全生产形势总体平稳。继续加强社会治安综合治理，不断完善公共安全管理手段和措施，集中力量开展严打整治行动，始终保持对刑事犯罪的高压态势，“平安朝阳”建设扎实推进。民兵预备役建设、基层武装、兵员征集和退役安置工作实现历史性突破，双拥和军民协作区建设取得重大进展。

——狠抓政府自身建设。坚持依法行政，深化政府自身改革和体制机制创新，推进政府职能和作风转变，全面开展行政权力运行制度建设，各级政府驾驭市场经济能力、社会管理能力和服务能力不断提高。强化审计和监察，工程建设领域突出问题和“小金库”专项治理取得明显成效。深入开展反腐倡廉，违纪违法案件得到查处。自觉接受人大及其常委会的法律监督和工作监督，主动接受政协的民主监督，积极支持各民主党派、工商联、无党派人士和人民团体参政议政，共办理人大代表议案、建议和政协提案218件，办复率和办结率均达到100%。

此外，质量监督、工商、物价、档案、住房公积金管理，民族工作和宗教事务管理，防震减灾、人防、外事侨务和涉台工作，以及老年人、妇女儿童、残疾人权益保障等方面也取得了新成绩。

各位代表，朝阳发展取得的每一项辉煌成就，都饱含着省委、省政府对我们的亲切关怀和大力支持，都凝结着全市各族人民的聪明才智、拼搏精神和辛勤汗水。实践有力地证明，只要我们紧紧抓住发展第一要务不放松，始终咬住发展目标不动摇，把上级的关怀支持充分转化为发展动力，最大限度地激发全市人民的创造力，形成万众一心共谋振兴的强大合力，就一定能够把朝阳的现代化建设扎实地推向前进。借此机会，让我们对多年来给予朝阳特殊关爱的省委、省政府表示最真诚的谢意！同时，我代表市人民政府，向为家乡的发展和振兴做出不懈努力和贡献的广大工人、农民、知识分子、各级干部、驻朝解放军指战员、公安干警和武警官兵，向依法实施监督，积极参政议政，鼎力支持和帮助政府正确履行职责的各位人大代表、政协委员，各民主党派、工商联、各人民团体和各界人士，向中央和省驻朝单位以及所有关心和支持朝阳发展的朋友们，表示衷心的感谢和崇高的敬意！

各位代表，我市当前和未来的发展还面临不少困难，政府工作仍存在一些问题。突出表现在：过分依赖资源的粗放型发展状况没有得到根本改变，扩张经济总量、转变发展方式任务仍十分紧迫；城镇化水平低，科技创新能力薄弱，驱动经济社会发展的动力不足；经济社会发展水平和人民群众收入水平，与全省还有较大差距；安全生产、社会稳定等方面的压力仍然较大；政府自身建设还存在薄弱环节，行政权力运行还不够规范，软环境建设有待进一步加强，个别干部还出现了违法违纪行为。各级政府必须高度重视这些问题，必须采取积极有效措施，加大力度予以解决。

“十二五”时期经济社会发展主要目标和重点任务



各位代表，我们已经进入第十二个五年规划期。未来五年，我们要以党的十七届五中全会精神为指导，深入贯彻落实科学发展观，抢抓机遇，坚定不移地坚持科学发展、创新发展、和谐发展和跨越式发展，坚持以增量带动结构调整，以创新促进产业升级，以发展保障民生改善，加快推进工业化、农业现代化、服务业多元化、城镇化和城乡生态化进程，不断提高综合经济实力，不断提高社会建设水平，不断提高人民生活质量，率先实现突破辽西北的战略目标，努力把朝阳建设成为东北地区重要的交通枢纽城市，辽冀蒙交汇区域最具发展活力的中心城市，我国重要的冶金、新型能源和绿色优质农产品基地，北方著名生态城市和文化旅游胜地。

根据市委的建议，市政府编制了《朝阳市国民经济和社会发展第十二个五年规划纲要（草案）》，已提交本次大会审议。今后五年，我们将按照纲要认真组织规划的实施，全力开创朝阳经济社会发展和现代化建设新局面。

——经济总量和综合竞争力再上新台阶。地区生产总值年均增长17%以上，财政一般预算收入年均增长22%，到“十二五”末，总量分别达到1800亿元和180亿元，均比“十一五”末翻一番半。

——城乡经济水平全面提升，工业主导地位更加凸显。在加快发展现代农业、现代服务业的同时，全面实施工业五项工程，继续做大做强冶金、装备制造、农产品加工、能源及新能源电器等重点产业，积极发展新兴产业，到2015年三次产业比达到14∶57∶29，实现向工业化中期迈进的历史性跨越。

——经济发展动力机制和支撑能力显著增强。强力推进项目建设，固定资产投资年均增长30%以上，五年累计突破7000亿元，比“十一五”翻两番。社会消费品零售总额年均增长18%，消费拉动经济增长能力不断增强。实际利用外资、出口创汇年均分别增长35%和20%。

——城市布局和基础设施更趋完善。燕都新城和朝阳县城建设初具规模，龙城新区、凤凰新城基本建成，“双百”城市目标基本实现；统筹城市交通、公共服务等基础设施建设，加快县城和小城镇建设，城镇化率达到50%以上。大力推进重点铁路工程、绥克高速公路、“一纵一环”公路以及电网、供热和天然气管道等项目建设，不断增强基础设施服务功能。

——生态建设和环境保护水平明显提升。大力实施治山治水工程，全面完成500万亩荒山造林和120万亩坡地造林任务，进一步巩固绿化成果，森林覆盖率达到45%；加快治理域内重点河流，确保大小凌河生态恢复工程取得成效。强力推进节能减排，有效控制环境污染，努力实现各类资源的合理开发和有序利用。

——人民群众生活不断改善。城镇居民人均可支配收入、农民人均纯收入年均分别增长16%和17%，大幅缩小与全省平均水平的差距。创造更多就业机会，年均新增就业岗位3.4万个以上。加强社会保障体系建设，不断提高保障水平。大力发展科技、教育、文化、卫生、体育等各项事业，全面提升社会文明和谐程度。

2011年重点工作安排



各位代表，今年是实施“十二五”规划的开局之年，做好今年工作，迈好“十二五”第一步，至关重要。我们要认真贯彻落实市委八届十二次全会精神，千方百计保持经济的良好走势和增长动力，千方百计推进各项事业发展，千方百计促进社会和谐稳定，确保各项重点工作在“十二五”开局之年旗开得胜、首战告捷。主要预期目标是：地区生产总值实现770亿元，增长18%；地方财政一般预算收入实现80.8亿元，增长22%；固定资产投资完成741亿元，增长30%；社会消费品零售总额完成254亿元，增长18%；城镇居民人均可支配收入和农民人均纯收入分别实现15500元和7140元，增长16%和17%。

（一）开展招商引资高峰年活动，全面提高对外开放水平

强力推进招商引资。坚持市政府主导推动、县（市）区为主实施的招商工作方式，以辽宁省内和国内外与我市产业关联度高、互补性强的城市为重点，放开视野，增加密度，突出重点，注重质量，搞好资源招商和产业链招商，加大农产品深加工、先进制造业、服务业、房地产开发等项目引进力度，确保每个月都有我市自主组织的大型招商活动，每个县（市）区都有鲜明的招商主题概念，每个重点行业都有重点招商项目，每个项目在引进和落地建设上都能达到高效率，争取域外引资额突破500亿元。同时积极组织对外招商和产品出口，力争实际利用外资增长35%，出口创汇增长20%。

积极争取中省直企业、域内外企业投资。继续加深合作，争取鞍钢、中电投等央企扩大投资，加快实施鞍凌精品钢、燕山湖电厂二期工程；加强跟踪推进，确保国电热电联产项目开工建设。积极创造条件，吸引更多中省直企业和域外大企业、大集团来朝投资。新建一批风力发电项目，新增50万千瓦装机容量。实施民营企业成长工程，鼓励和支持本地民营企业扩大投资、做大做强。积极开展全民创业活动。全年实施投资千万元以上重点项目650个。

多渠道引入各种资金。充分利用沿海经济带政策，加大投入力度，加快推进纳入政策支持范围园区的建设和发展。大力开发资本市场，通过引进股权基金、推进企业上市等措施，破解基础设施建设、中小企业融资难的瓶颈。优化金融生态环境，鼓励引导金融机构加大信贷投放。积极推进城市商业银行增资扩股，加快实现跨区域经营。完善金融体系建设，努力引进域外银行到我市开办金融业务，新增3家规模较大的小额贷款公司。

（二）以实施工业五项工程为重点，加快增强工业整体实力	按照省政府的统一部署，下大力量抓好工业五项工程。

提升企业规模。深入推进企业联合重组，积极引进战略合作伙伴，狠抓骨干企业培育，力争新增销售收入百亿元企业1户、超10亿元企业2户、超2000万元以上企业20户。

壮大产业集群。加快新能源电器产业基地建设，新建标准厂房4.3万平方米，新增投资50亿元以上。北票粉末冶金和除尘装备、朝阳县有色金属产业集群产值分别突破150亿元和130亿元，其他产业集群产值增长35%以上，并加快向百亿元目标迈进。

推进重点工业项目建设。全力支持凌钢实施扩能改造和产品升级，并带动一批相关企业发展；加快鞍凌精品钢和燕山湖电厂一期、普耐特500万条乘用胎、柏慧燕都肉制品加工、新浙锰业中低碳锰、红山玉板材等项目投产达产；积极推进太阳能光伏产业链、北票和喀左太阳能发电、凌源鸿锐曲轴、龙城混合动力发动机、双塔航天电子城等项目建设；加快推进浪马、朝柴扩大产能及新产品研发。

促进科技创新和企业并购。积极设立院士专家工作站和博士后工作站；引领20家企业与高校院所建立产学研合作关系；市级以上企业研发机构达到80家。全市高新技术产品增加值增长25%以上。启动企业并购项目10户，完成并购3户。

狠抓节能降耗和淘汰落后产能。确保万元工业增加值能耗和用水量、固体废物综合利用率及淘汰落后产能完成省下达指标。

在抓好五项工程的同时，继续加大结构调整力度，充分利用区域资源和产业基础，接长产业链条，搞好精深加工，提高产品附加值，推动传统产业优化升级。以生物制药、新材料、节能环保等为重点，培育和发展新兴产业。积极搭建企业家培训平台、中小企业融资平台，强化对企业的服务。采取有效措施，加大对中小企业的扶持力度。加强工业经济运行调度，保障重点骨干企业生产顺利进行，确保规模以上工业增加值增长20%以上。

（三）坚持双轮驱动，加快县域经济和城区经济发展

壮大县域经济规模。以县域经济为载体，深入实施“五个一工程”，大力推进工业集群化、农业产业化和农村城镇化，加快新农村建设。启动新一轮县域经济三年倍增计划，全力支持北票、建平向全国百强县（市）迈进，其他县（市）朝着地区生产总值超百亿元、财政一般预算收入超十亿元目标努力。

继续抓好粮食生产。全面完成100万亩设施农业建设任务，加快棚内种植结构调整，下大力量抓好种苗培育、市场建设、技术和管理培训环节的衔接，确保取得良好的经济效益。新增800个畜禽标准化养殖小区，搞好重大动物疫病防控工作。加快形成一县一业特色农业格局，增强市场竞争力。继续支持农业产业化龙头企业做大做强，积极引进高附加值企业，重点扶持投资超10亿元的龙头项目。大力推进标准化生产，加快培育有机绿色无公害品牌。深入推进农业专业合作社建设，稳定和壮大专业技术人员队伍。

加快城区经济和服务业发展。做大做强双塔、龙城两区经济规模，全面实现第一轮三年倍增目标。积极做好市经济技术开发区扩区工作，为进入国家级开发区行列做好准备。

坚持生产性和生活性服务业并举、改造提升传统服务业与做大做强新兴服务业并行，加快服务业多元化进程。重点推进中央商贸服务业聚集区、龙城现代服务业聚集区和文化旅游特色产业带开发建设，支持各县（市）区规划建设销售收入超50亿元的服务业聚集区。依托区位和产业优势，积极促进大型物流基地建设。不断创新财税管理方式，提高税收征管水平和理财能力。大力加强各级财源建设，加快可支配财力增长，增强政府调控能力。

（四）加大城乡建设力度，加快推进城镇化

全力推进燕都新城建设。年内力争完成10平方公里核心区基础设施建设，启动实施市行政中心、第四人民医院等一批公建项目和商业开发项目，加快殡仪馆、康宁医院等公共服务设施搬迁，确保新城形成初步形象。同步加快龙城新区和凤凰新城建设。继续推进城区道路拓宽、热网和燃气管道改造项目。

加快县城和重点小城镇建设。全力推进朝阳县城建设，完善道路、电力、给排水等基础设施，开工建设县行政中心大楼。其他县城要进一步扩容提质，在建成区规模和建设品位上实现突破。精心搞好5县（市）5镇2村的城镇化推进试点工作。以大小凌河综合治理为契机，全面启动沿岸珍珠式新市镇规划。

推进城乡基础设施建设。积极配合京沈高铁、锦赤铁路新线、锦承线改造扩能、牛河梁铁路改造及秦沈线天然气管道支线建设，扎实做好绥克高速公路项目争取工作，启动实施“一纵一环”公路项目。大力推进500千伏利州输变电和新一轮农网升级改造。加快推进凌源、建平城区应急供水工程和大凌河城区段三期工程。

稳步推进房地产业和建筑业。全市完成房地产开发800万平方米、城中村改造100万平方米。实现建筑业产值160亿元。

加大城市管理力度。各级政府都要重视城市管理。中心城区要充分发挥街道、社区、门前“三包”单位作用，建立有效的激励和约束机制，实现城市管理重心下移。加大综合治理力度，扩大管理覆盖面，强化管理手段，打造文明、整洁、优美、有序、安全的城市环境。积极推广喀左“五城联创”经验，加速实现各县城的绿化、净化、美化和亮化。

（五）加强生态建设和环境资源保护，增强可持续发展能力

扎实推进造林绿化工作。坚持经济效益和生态效益并重，全面完成500万亩荒山造林任务；高质量推进45万亩年度坡地经济林造林工作。加强自然保护区建设和管理，狠抓封山禁牧和森林防火，提高矿山植被恢复率，巩固生态建设成果。

加强水资源保护和利用。积极推进大小凌河、老哈河、青龙河流域综合治理，搞好河道防洪体系建设和病险水库除险加固工作，加快恢复河道生态环境。继续开展水土保持综合治理，完成治理面积42万亩。积极开展人工影响天气工作，完善增雨设施，科学开发云水资源，积极发展节水型农业。

认真落实节能降耗减排措施。树立低碳理念，大力发展循环经济，深入推进“创模”工作，确保节能减排全面完成国家和省定目标。坚持土地资源节约集约利用，充分利用增减挂钩政策，加大用地指标争取力度，保证项目用地需求。加大矿产资源勘查力度，持续整顿和规范矿产资源开发秩序。

（六）大力发展社会事业，着力保障和改善民生

提升教育发展水平。统筹城乡和各级各类教育，整合资源，协调发展，促进教育公平。新建市县两级标准化幼儿园20所，逐步解决学前儿童入园难问题；深入推进义务教育均衡发展示范县区创建工作，确保建平县通过省“双高普九”评估验收；完成市二中易地新建主体工程；加快普通高中优质特色化建设；大力发展职业教育，为我市工业化发展提供技能型人才，促进校企合作，提高企业技术创新、管理创新能力；完成师专搬迁工作，推进申办本科大学进程；下力量解决好农村中小学教育布局调整后部分教育资源闲置问题。继续抓好民族教育和民办教育。

改善城乡医疗卫生条件。加快城乡公共卫生服务体系建设，完成市中心医院外科综合楼、市二院病房楼主体工程，搞好7个社区卫生服务中心和25个标准化村卫生室建设。继续扩大新农合覆盖面，常住人口参合率保持在99%。完善重大疾病防控体系和人员聚集场所公共卫生保障机制，提高应急处置能力。强化食品、药品生产销售监管，保障人民群众饮食用药安全。

繁荣文化事业和文化产业。深入实施文化惠民工程，新建标准乡镇综合文化站82个。完成牛河梁二号地点保护展示馆和重点基础设施建设，扎实推进“申遗”工作；全面完成鸟化石国家地质公园二期工程，启动世界地质公园和自然遗产申报工作；以南北塔文物博览园区、凤凰山旅游产业示范区、清风岭旅游区、温泉休闲度假区建设以及乡村旅游资源开发为重点，加快文化旅游基地建设，进一步做好文化遗产抢救、挖掘和保护工作。

同步推进其他各项事业。深化全民健身运动，提升竞技体育水平，高质量承办第六届全省农运会；继续完善体育设施，促进体育产业发展，完成全民健身中心规划，建设50处农民体育健身工程。坚持不懈地抓好人口和计划生育工作，稳定低生育水平，提高人口素质。优化人才环境，加大人才培养和引进力度。认真落实党的民族政策，支持民族地区加快发展，增进民族团结。加强国防后备力量建设，抓好兵员征集和军转干部、退役士兵安置，深入开展以军民协作区建设为主要内容的双拥工作，努力争创全国双拥模范城。

全力抓好就业、社会保障和扶贫工作。加强困难群体就业援助，完成实名制就业4.5万人，城镇登记失业率控制在4.5%以内。建立健全职工工资正常增长机制、支付保障机制以及工资集体协商制度，不断提高职工实际收入水平。积极构建和谐的劳动关系，保障劳动者合法权益。继续扩大各类社会保险覆盖面，完善城乡低保制度，提高保险保障水平，同时搞好社会救助、减灾救灾工作。完成整村推进项目村100个、移民搬迁700户。

维护社会安全稳定。建立健全社会稳定风险评估机制，注重矛盾纠纷源头预防和治理，依法做好信访工作，积极解决群众合理诉求，努力把矛盾化解在基层。深入开展平安创建活动，推进社会管理创新，全面加强治安综合治理，严厉打击违法犯罪行为，全力打造平安朝阳、和谐朝阳。加强安全生产监管和专项整治，严防重特大事故发生。完善应急管理机制，提高防范和处置各种突发公共事件的能力。加强价格监管，强化平抑手段，维护物价稳定。加强社会主义民主建设和法制建设，促进社会公平正义。

为人民群众办好10件实事。一是提高城乡居民最低生活保障标准。二是提高全市重点优抚对象抚恤补助标准。三是在五县（市）实现新型农村养老保险全覆盖。四是提高城镇职工基本医疗保险待遇标准。五是解决15万农村人口饮水安全问题。六是对设施农业大棚实施政策性保险。七是市县两级实施100万平方米老旧小区环境改造工程。八是全市新建廉租房6万平方米、经济适用房8万平方米。九是提高企业退休人员养老金标准。十是解决100个偏远自然村移动信号覆盖问题。

（七）加强政府自身建设，依法规范有序地履行职责

各级政府必须不断强化自身建设，积极转变职能、转变作风、转变服务方式，全面适应新形势、新任务和新要求。要深入解放思想，更新观念，从实际出发，做开拓性工作。要勇于面对困难和矛盾，敢做敢为，大胆负责，以科学的方法、有力的举措、扎实的作风抓好各项工作落实。要创新管理机制和手段，有效解决机关当中存在的慢作为、不作为、乱作为问题，保证工作高质量、快节奏推进。要不断强化法制观念，自觉依法行政，全面提高行政执法水平。要进一步深化政务公开，完善科学民主决策机制，在自觉接受人大及其常委会法律监督、工作监督和政协民主监督的同时，广泛接受社会各界各阶层及广大群众的监督，充分尊重公众的知情权、表达权、参与权，虚心听取采纳各方面的意见和建议。要严格落实党风廉政建设责任制，加强行政权力运行制度和惩防体系建设，加大执法监察、审计监督和行政问责力度，严肃查处各类违法违纪案件。要继续深化行政审批制度改革，加强机关效能和行风建设，下大力量改善经济发展软环境。通过采取一系列措施，努力把各级政府建设成为求真务实、真抓实干、勤政为民、清正廉洁的政府。

各位代表！告别了不平凡的“十一五”，朝阳发展步入了新的历史征程。尽管前进的道路并不平坦，还会遇到种种困难和挑战，但这阻挡不了朝阳加快崛起的步伐！让我们携起手来，在市委的坚强领导下，充分发扬朝阳精神，万众一心，众志成城，为实现“十二五”新的更大的发展，为早日完成突破朝阳、振兴朝阳的宏伟大业，为朝阳人民不断过上更富裕、更有尊严的生活而努力奋斗！
